# Author Correction: Heat acclimation in mice requires preoptic BDNF neurons and postsynaptic potentiation

**DOI:** 10.1038/s41422-025-01073-z

**Published:** 2025-01-24

**Authors:** Baoting Chen, Cuicui Gao, Changhao Liu, Tongtong Guo, Junwei Hu, Jialiang Xue, Kangmin Tang, Yuelai Chen, Tian Yu, Qiwei Shen, Hongbin Sun, Wen Z. Yang, Wei L. Shen

**Affiliations:** 1 School of Life Science and Technology & Shanghai Clinical Research and Trial Center, ShanghaiTech University, Shanghai, China; 2 Longhua Hospital, Shanghai University of Traditional Chinese Medicine, Shanghai, China; 3 Shanghai University of Traditional Chinese Medicine, Shanghai, China; 4 Guizhou Key Laboratory of Anesthesia and Organ Protection, Zunyi Medical University, Zunyi, Guizhou China; 5 Department of General Surgery, Huashan Hospital, Fudan University, Shanghai, China

**Keywords:** Molecular biology, Cell biology

Correction to: *Cell Research* 10.1038/s41422-024-01064-6, published online 26 December 2024

In the original publication of this paper,^1^ the funding number for the National Natural Science Foundation of China to W.L.S. was incorrect. The funding number listed as 9235730017 should be corrected to 92357304. We apologize for this carelessness.

The original article has been corrected.
